# Personalized *in situ* bioprinting of skin: designing a blueprint for clinical translation

**DOI:** 10.3389/fbioe.2026.1895767

**Published:** 2026-10-12

**Authors:** Rana Elmaalawy, Abdulrahman Khaled, Mona Marei, Rania Elbackly

**Affiliations:** 1 Tissue Engineering Laboratories, Faculty of Dentistry, Alexandria University, Alexandria, Egypt; 2 School of Mechanical & Materials Engineering, University College Dublin, Dublin, Ireland; 3 Prosthodontics Department, Faculty of Dentistry, Alexandria University, Alexandria, Egypt; 4 Endodontics, Conservative Dentistry Department, Faculty of Dentistry, Alexandria University, Alexandria, Egypt

**Keywords:** artificial intelligence, in situ 3D bioprinting, personalized medicine, skin tissue engineering, wound healing

## Abstract

*In situ* 3D skin bioprinting offers a revolutionary paradigm for directly regenerating massive cutaneous defects right on a patient’s wound bed. However, translating this technology from the bench to the bedside remains heavily bottlenecked by hostile wound microenvironments, delicate cell viability constraints, and unpredictable tissue deformation during surgery. To overcome these primary hurdles hindering its clinical translation, this review proposes a comprehensive blueprint for integrating artificial intelligence (AI) into the *in situ* bioprinting of skin. It describes how AI can optimize bioinks, design patient-specific grafts from medical images, and enable real-time adaptive printing through predictive modeling and feedback. The review presents a framework for developing intelligent, portable systems for personalized skin therapy while addressing biomaterials, personalization, quality control, and AI-related ethical issues. This roadmap ultimately bridges the gap between delicate laboratory protocols and autonomous, precision-guided intraoperative interventions tailored for real-world clinical deployment.

## 1 Introduction

The skin is a crucial barrier that prevents water loss and defends the body against mechanical injury, chemical irritants, and microbial pathogens. However, skin can become severely compromised or lost entirely due to massive structural damage in thermal burns, ischaemic or diabetic ulcers, as well as due to a myriad of skin diseases (Proksch et al., 2008). Indeed, skin and subcutaneous diseases impose a staggering global burden, with 4.69 billion incident cases in 2021 resulting in 41.9 million disability-adjusted life years (DALYs) (World Health Organization, 2025). Burn injuries similarly compromise the skin barrier on a massive scale, affecting 12.99 million individuals with severe burns and 235.34 million with mild burns in 2021; alarmingly, severe burn cases are projected to surge by 233.4% to reach 43.31 million by 2050 (Lee et al., 2025). Compounding these wound healing challenges is the escalating diabetes epidemic—a primary driver of severe skin compromise through diabetic ulcers—which affected 589 million adults in 2024, caused over 3.4 million deaths, and left 252 million individuals undiagnosed and vulnerable to complications, with overall prevalence projected to reach 853 million by 2050 (Genitsaridi et al., 2026).

Current treatments often fail to achieve complete regeneration or replicate the wound environment, driving interest in bioprinting. 3D bioprinting offers a highly promising solution, enabling the precise, automated spatial deposition of diverse cell types, biomaterials, and bioactive molecules to construct physiologically accurate, multi-layered human skin equivalents (Perez-Barreto et al., 2026).

*In situ* 3D bioprinting is defined as the direct printing of living cells, growth factors, and biomaterials to create or repair living tissues or organs at a defect site (Dong et al., 2023). The process of *in situ* bioprinting initiates with preprocessing, which involves imaging the target tissue or organ using techniques such as computed tomography (CT), magnetic resonance imaging (MRI), or ultrasound. These images are then used to reconstruct three-dimensional (3D) models. The generated 3D models are subsequently converted into the STL file format, which is the standard file format accepted by most commercially available bioprinters (Blaeser et al., 2016).

The material used for bioprinting is known as bioink, a substance that contains cells along with various bioactive components and supportive materials (Groll et al., 2019). Hydrogels are typically used as the main ingredient in these bioinks because their high water content allows them to effectively carry and maintain cells during printing (Malda et al., 2013). Bioinks are stabilized through physical, ionic, or chemical crosslinking. Physical crosslinking relies on non-covalent interactions like hydrogen bonding, ionic interactions, or polymer entanglement and is mild and cell friendly. Ionic crosslinking, common with alginate, uses divalent cations (e.g., Ca^2+^) to form gels. Chemical crosslinking forms covalent bonds, often via UV light or enzymatic reactions, providing stronger and more stable constructs (Gillispie et al., 2020; Panwar and Tan, 2016).

For dermatological applications, *in situ* bioprinting employs robotic arms or handheld devices to apply bioinks directly into irregular wound sites. Artificial intelligence and machine learning improve this process by automating image segmentation and bioink optimization while enabling real-time monitoring. These advances improve precision and reproducibility, bringing personalized skin repair closer to clinical use (Perez-Barreto et al., 2026). This review examines the current obstacles in *in situ* skin bioprinting and explores how overcoming technological and ethical barriers can accelerate its clinical adoption (Li et al., 2024) (Figure 1).

**FIGURE 1:**
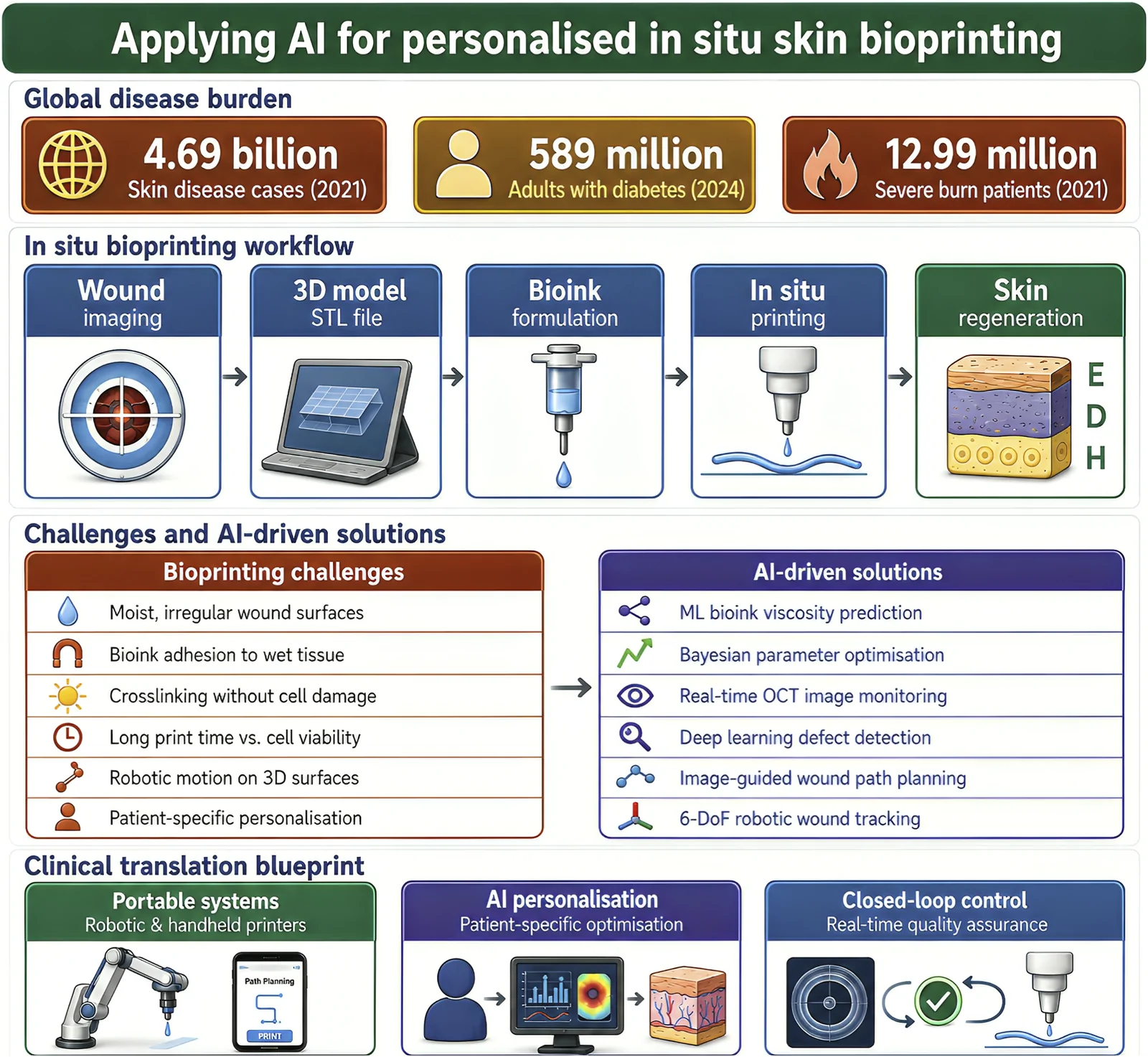
Illustration showing key aspects of implementing personalized *in situ* skin bioprinting.

## 2 Transforming theory into practice for 3D bioprinting of skin tissue equivalents in clinical settings

### 2.1 Challenges in *in situ* skin bioprinting

Skin bioprinting faces numerous challenges due to the sophisticated hierarchical structure of native skin, which emerges from multiple differentiated layers with distinct biological and mechanical properties. Another important requirement is that bioprinted skin possesses mechanical strength and elasticity comparable to normal skin, particularly when it is intended to cover highly mobile areas such as joints (Gupta et al., 2025) *Ex vivo* bioprinting presents additional challenges, including graft fixation, prolonged *in vitro* incubation, and limited customization during transplantation (Hu et al., 2024).

While *in situ* skin bioprinting may offer patient-specific solutions to large critical skin defects, it still poses significant challenges. Bioinks must combine mechanical strength, high adhesion, and rapid solidification to conform to moist, irregular wound surfaces. Multilayered printing also requires careful *in situ* crosslinking to avoid cell damage, and large defects extend printing time, further threatening cell viability (Chimene et al., 2016). The following sections discuss these challenges in detail.

#### 2.1.1 Challenges in the wound bed environment

For *in situ* skin bioprinting, the wound environment presents extremely challenging conditions. The tissue surface is often moist and irregular, containing blood, interstitial fluid, and exudates, which interfere with bioink deposition and adhesion. Many bioinks have limited mechanical strength immediately after printing, making constructs prone to deformation, collapse, or fragmentation. Such instability not only compromises the intended tissue architecture but risks damaging surrounding healthy tissue or releasing debris that could trigger inflammation or infection (Singh et al., 2020; Ding and Chang, 2018).

To address this challenge, a research group developed the first commercial articulated collaborative *in situ* bioprinter for the treatment of full-thickness skin wounds. This system integrates an articulated, collaborative robotic arm with a custom-designed printhead and dedicated software, enabling precise *in situ* bioprinting on curved and dynamically moving surfaces. *In vitro* and *in vivo* studies showed that the system achieved strong bioink adhesion, high structural fidelity, and reliable printing on wet, irregular tissues (Levin et al., 2022).

Other researchers have focused on optimizing the bioink itself to enhance adhesion for skin *in situ* bioprinting. They developed bioinks that allowed stable extrusion in wet environments and adhered well to chemically diverse surfaces (Li W. et al., 2023).

#### 2.1.2 Structural fidelity and cell viability

Maintaining the shape and structural integrity of multilayered skin constructs requires careful control of bioink solidification during printing. Indeed, it is essential that each layer be at least partially crosslinked before depositing the next (Knowlton et al., 2017). One of the most used crosslinking methods for *in situ* bioprinting involves ultraviolet (UV) irradiation. However, in skin-related applications, excessive UV exposure can damage cells. To address this limitation, several approaches have been proposed (Xu et al., 2022).

One study investigated a visible light–based system for photo crosslinking bioprinted scaffolds. Although blue light (405 nm) is close to the UVA spectrum, it exhibits significantly lower cytotoxic effects. In this study, scaffolds were printed in a layer-by-layer manner and crosslinked using either a conventional UV system or a visible light system. Constructs polymerized under visible light demonstrated markedly higher cell viability than UV-crosslinked scaffolds, maintaining over 80% cell survival even at elevated photoinitiator concentrations. These results indicated that visible light was a safer and effective alternative for *in situ* bioprinting (Lim et al., 2016).

Another approach utilized a microfluidic printhead with a two-step crosslinking strategy for *in situ* bioprinting. During printing, an ionic crosslinker was co-deposited with the bioink to provide immediate mechanical support. After the complete structure was fabricated, the whole construct was covalently solidified using UV light. This strategy can enable the fabrication of accurate multilayered structure with strong interlayer bonding while reducing the risk of cell damaging that can happen with prolonged exposure to UV (O’Connell et al., 2020; Colosi et al., 2016).

In order to overcome the crosslinking challenges, researchers have developed smart bioink materials with responsive or adaptive properties that can change their behaviors in response to external stimuli or the surrounding biological environment thus introducing the concept of 4D bioprinting (Amukarimi et al., 2022; Kim and Cho, 2024). These bioinks respond to temperature, pH, light, and magnetic or electric fields to improve stability and enable precise structuring (Amukarimi et al., 2022). Such responsiveness makes smart bioinks highly versatile, allowing improved control over printing processes and material characteristics.

Montero et al. (2019) developed and thoroughly characterized a smart bioink, which exhibited thermoresponsive behavior, with solution temperatures around 34 °C–35 °C, making it well-suited for physiological conditions. Rheological analyses demonstrated that all formulations were pseudoplastic, ensuring compatibility with extrusion-based 3D bioprinting strategies.

#### 2.1.3 Bioprinting of large critical size skin defects

The treatment of large skin defects remains a major challenge for *in situ* bioprinted skin constructs. Such defects require rapid intervention to minimize the risk of infection and excessive inflammation. To overcome this limitation, Zhao et al. created a novel bioink based on platelet-rich plasma (PRP) which showed better wound healing results and stronger cellular attachment and faster development of customized tissue (Zhao M. et al., 2022).

Additionally, the extended surgical and printing procedures associated with *in situ* bioprinting of large defects could negatively affect the cells. This long exposure of cells to varying shear stresses, prolonged temperature fluctuations, and crosslinking might be detrimental to cell health (Douglas et al., 2025; Malekpour and Chen, 2022). The operation requires specific control of these elements to maintain cell viability during extended operational times (Xu et al., 2022).

Among these factors, shear stress is one of the most critical parameters influencing cell survival. Cell viability remains above 90% when shear stress is below 5 kPa. However, higher extrusion pressures and smaller nozzles increase cell damage despite improving print quality. Several researchers have used computational fluid dynamics (CFD) to develop bioprinter nozzle designs which decrease shear stress on bioinks while enhancing cellular treatment success (Gómez-Blanco et al., 2023; Magalhães et al., 2019). These studies consistently report that increased shear stress correlates with reduced viable cell populations. The duration of exposure to shear stress plays a crucial role in determining cell survival during extrusion (Manzoli et al., 2025; Gómez-Blanco et al., 2020). Wei et al. (2024) conducted CFD simulations to investigate the influence of nozzle geometry on flow-induced forces and cell viability by comparing conical and cylindrical nozzles. Their findings demonstrated that conical nozzles reduce exposure time to damaging shear stress and are therefore more biocompatible than cylindrical designs.

In a related study, Lombardi et al. (2025) created a custom nozzle that integrates with their current bioprinter system to improve cell survival rates. Their model clarified how nozzle geometry influences stress-induced cell damage and can guide future nozzle designs. The studies show that researchers have dedicated their efforts toward developing new printing machines which will decrease the physical strain on equipment.

In parallel, other studies have focused on modifying printing hardware to reduce mechanical stress. Yumoto et al. (2020) used an inkjet bioprinter with a piezoelectric inkjet head to create a system that effectively reduces shear stress during printing. The system controlled internal pressure using a disk-membrane nozzle and an open chamber with a bending-type piezoelectric actuator.

Temperature regulation is essential for maintaining both printability and cell viability, particularly when using thermosensitive hydrogels such as gelatin methacrylate (GelMA). Wang Z. et al. (2024) developed a PID-controlled printhead that maintains a constant bioink temperature during printing. The method maintained consistent material flow characteristics which resulted in uniform filament deposition and stable scaffold design.

Moreover, crosslinking methods significantly affect cell viability. Studies have shown that ionically crosslinked materials can achieve high cell viability without requiring light exposure (Choi et al., 2019). However, photocrosslinking is still widely used, and recent innovations aim to mitigate associated phototoxic effects. For example, Duchi et al. (2017) introduced a handheld coaxial bioprinter (the “Biopen”) to minimize phototoxicity. The Biopen extrudes two concentric layers: a GelMA core containing cells without photoinitiator and an outer shell containing Lithium phenyl-2,4,6-trimethylbenzoylphosphinate (LAP), which is crosslinked by UV light during printing. This configuration shields embedded cells from direct light exposure while enabling rapid scaffold stabilization, resulting in cell viability exceeding 95%.

Finally, insufficient oxygen and nutrient diffusion continue to be a significant obstacle for constructing large and thick skin structures. Scientists discovered a solution to this problem by studying salamanders, which maintain oxygen supply through their photosynthetic algae. The natural symbiotic relationship between organisms has led to the use of living microalgae (Chlorella pyrenoidosa) as a component in bioprinted scaffolds. The unicellular algae use light to perform oxygenic photosynthesis, which creates a continuous oxygen supply that exists in their environment. The scaffold operates as a biological system that generates oxygen, which helps to treat hypoxia while supporting blood vessel formation, thus helping tissue regeneration to progress (Wang et al., 2022).

These aforementioned strategies may represent potential methods to help cells stay alive while they maintain their natural structure throughout the bioprinting process.

### 2.2 Methods for bioink deployment during *in situ* skin bioprinting

The three main approaches that can be utilized for *in situ* bioprinting are Droplet-Based Bioprinting (DBB), Extrusion-Based Bioprinting (EBB), and Laser-Based Bioprinting (LBB) (Huang et al., 2025).

Droplet-Based Bioprinting (DBB) (Sun et al., 2020) functions by ejecting small droplets of bioink through a nozzle using thermal, piezoelectric, or acoustic forces. Each droplet contains a mixture of cells and biomaterials that are deposited layer by layer to form a three-dimensional structure. This method is conceptually similar to inkjet printing and offers high precision in droplet placement.

Laser-Based Bioprinting (LBB) (Dou et al., 2021) employs focused laser energy to transfer bioink droplets from a donor layer onto a receiving substrate with precise spatial control. The laser pulse generates localized pressure that propels the bioink droplets toward the target, enabling highly accurate cell placement without direct mechanical contact.

Extrusion-Based Bioprinting (EBB) (Ozbolat and Hospodiuk, 2016) utilizes a robotic system to continuously extrude bioink as filaments through a nozzle. The extrusion process can be driven by pneumatic, piston, or screw-based mechanisms.

Techniques such as DBB and LBB still lack extensive clinical application in *in situ* bioprinting. In contrast, extrusion-based bioprinting (EBB) continues to be the dominant printing method because it offers both flexible printing options and the ability to work with highly viscous bioinks that contain numerous cells. This is exemplified by a highly advanced handheld, programmable bioprinter used to fabricate skin wound dressings directly onto large wounds in animal models. The system incorporates a web app interface, allowing users to operate the bioprinter via smartphones. This development represents a significant step toward scaling up these technologies for clinical implementation (Wang C. et al., 2024).

Each of these methods has its own advantages and limitations, making it challenging to determine the most suitable technique for specific bioprinting applications (Table 1).

**TABLE 1:** Shows the advantages and disadvantages of different techniques of *in situ* bioprinting (Huang et al., 2025).

Bioprinting technique	Pros	Cons
1. Droplet-Based Bioprinting (DBB)	• High speed • High resolution • Good cell survival (80%–90%)	• Requires low-viscosity bioink (needs additional crosslinking step) • Uneven droplet sizes • Nozzle clogging when using dense bioinks
2. Extrusion-Based Bioprinting (EBB)	• Can print thick or highly viscous bioinks • Allows multiple print heads for complex structures	• Lower cell survival (40%–86%) due to high pressure and shear stress
3. Laser-Based Bioprinting (LBB)	• No nozzle (prevents clogging) • Minimal cell damage • Very high precision	• Still an emerging and less widely adopted technique

### 2.3 Clinically applicable strategies for *in situ* skin bioprinting

There have been two main approaches employed for *in situ* bioprinting. These include the use of handheld bioprinters or robotic assisted bioprinting or a combination of both approaches (Figure 2).

**FIGURE 2:**
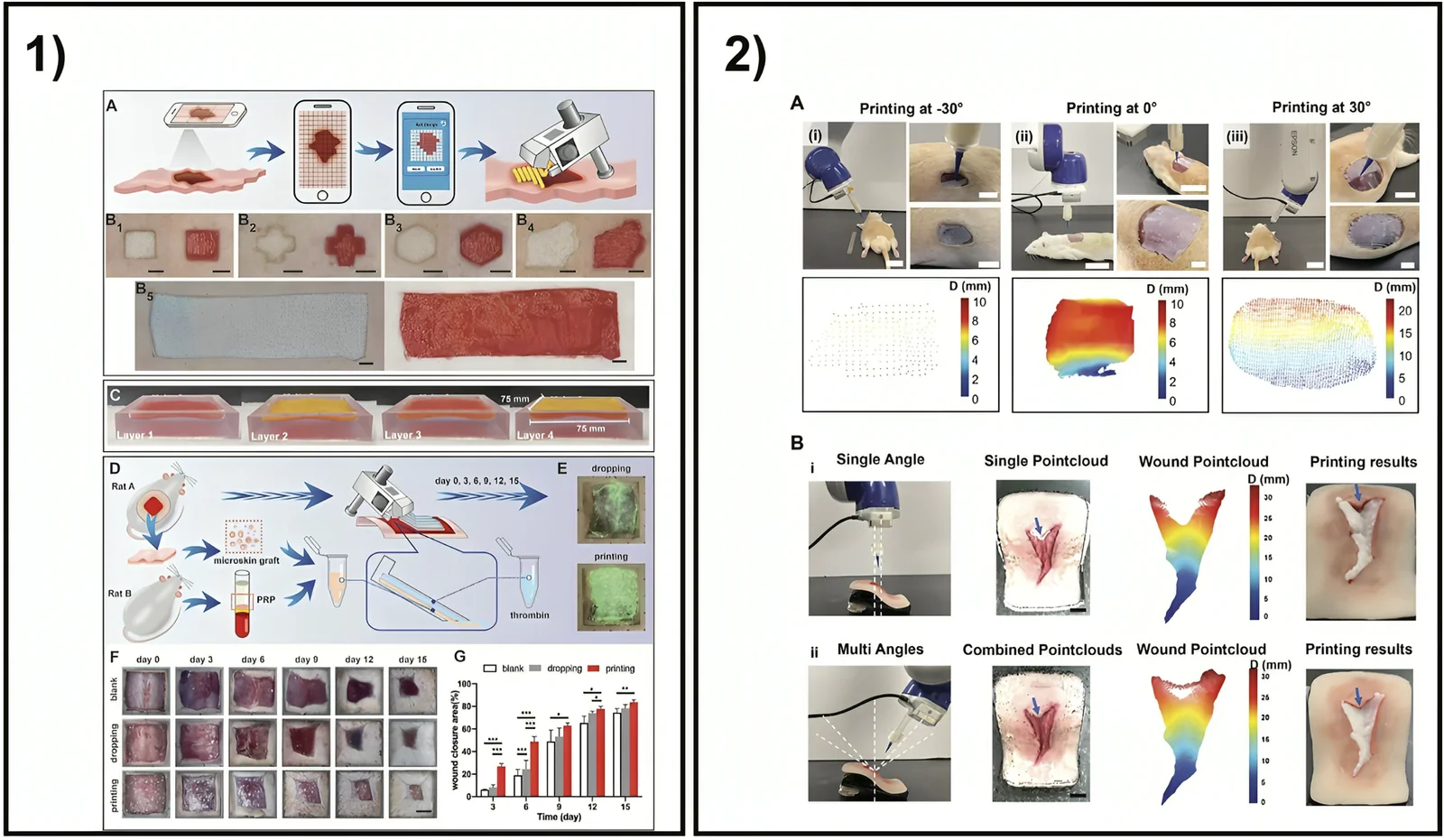
Robotic bioprinting system for in situ wound treatment. 1) Programmable handheld in situ bioprinting system for skin wound treatment, demonstrating image-guided wound coverage across various wound shapes on porcine skin, multi-layer printing using a microchannel nozzle, and in vivo validation in a rat full-thickness wound model using PRP-microskin graft bioinks, with improved wound closure compared to conventional droplet delivery. 2) In situ bioprinting at tilted orientations (–30°, 0°, and +30°), with 3D point cloud acquisition demonstrating that multi-angle scanning recovers complex wound geometries missed by single-angle approaches (blue arrows). Figures are reproduced from articles (Wang C. et al., 2024; Jeong et al., 2024; License number 6352941184625 from John Wiley and Sons).

#### 2.3.1 Robotic-assisted bioprinters

An *in situ* robotic bioprinter needs to define its operational area but it requires imaging systems to locate its working zone and perform bioink printing through predetermined paths of movement (Dong et al., 2023) To dispense the ink, the bioprinter nozzle must move, and several motion strategies exist to facilitate printing. The most conventional strategy is the Cartesian or linear robot configuration. In this method, the nozzle’s motion is restricted to a two-dimensional plane along the printing axes (X,Y,Z). While this is the simplest and lowest-cost method, its operation is non-flexible due to its axes-restricted movement, making it ineffective for printing on curvilinear or irregular surfaces (Dong et al., 2023).

To address the limitations of Cartesian systems on irregular surfaces, the second type of bioprinter robot is the articulated robot. An articulated robotic arm is a human-like structure with a motorized configuration that can rotate freely. Most systems feature three to six axes, which allows the printing nozzle to approach curved or irregular surfaces from virtually any direction. This multi-axis design grants articulated robots significantly greater flexibility, mobility, and spatial reach, making them especially useful for complex *in situ* bioprinting tasks (Li K. et al., 2023).

A significant example utilizing the articulated robotic arm model is the da Vinci Arms (Bodner et al., 2005). Initially designed to enable remote human-controlled surgery, this technology has progressed toward highly automated robots capable of performing surgery with only human supervision. This system may be applied later to *in situ* bioprinting.

Another specific type of articulated robotic arm is the Selective Compliance Articulated Robot Arm (SCARA). Its parallel-axis joints provide flexibility in the X–Y plane while maintaining rigidity along the Z-axis, making it suitable for 3D bioprinting applications requiring controlled directional movement (Li K. et al., 2023).

Finally, parallel robots typically have multiple arms (usually three connected to a single base mounted above the workspace). These robots often employ mechanisms like articulated robots for movement, and they are known for delicate and precise motion. Parallel configurations share many of the advantages and disadvantages of articulated robots, but they introduce an additional potential issue. Singularity occurs when the nozzle becomes temporarily stuck in one position due to a motion error or specific joint alignment (Li K. et al., 2023). However, other advantages of the parallel configuration include a simple structural design and easy installation. A major innovation within this type is the Delta robot (Zhao and Xu, 2020), which can fold itself into a smaller size for insertion into a patient’s body and then unfold before bioprinting, making it particularly useful for minimally invasive procedures. These robotic arms are a promising option in skin bioprinting.

Early attempts at *in situ* skin bioprinting utilized in-house designed computer-controlled bioprinters to treat large skin wounds in mice using amniotic fluid-derived stem cells or bone marrow-derived mesenchymal stem cells resuspended in fibrin-collagen gels displaying effective healing outcomes (Skardal et al., 2012). While such studies mainly focused on the role of the cells in the treatment strategy, they introduced the potential of *in situ* bioprinting as a potential off-the-shelf rapid method to manage large critical skin wounds.

These findings led to more advanced systems, including a mobile robotic skin bioprinter that combines a handheld 3D wound scanner with an XYZ-controlled printhead and eight independently actuated nozzles. The system is designed to be compact and portable, allowing use within an operating room while maintaining sufficient reach to cover large body areas such as the torso (Albanna et al., 2019). A portable *in situ* 3D skin bioprinter integrating a three-degree-of-freedom robotic arm and an advanced scanning module to capture wound geometry and reconstruct a customized 3D wound model. The system could achieve filament deposition with cell survival rates exceeding 89%–95% (Zhou et al., 2024).

The potential of robot-assisted *in situ* bioprinting has also been recently demonstrated in a murine skin wound model. Different sized full-thickness skin wounds were created on the backs of the mice and transplanted with appropriate volume 2.5% or 5% gelatin methacrylate (GelMA) hydrogels contained epidermal stem cells (Epi-SCs) and skin-derived precursors (SKPs) by pipet or robotic-assisted *in situ* bioprinting under light exposure. Results after 4 weeks displayed the regeneration of complete skin with appendages closely resembling healthy skin. Adopting such a strategy in a clinical scenario may offer the advantage of reconstructing complex defects in real-time and respect the native tissue microenvironment (Chen et al., 2023a). A pilot study used a customized 6-axis robotic arm to treat third-degree burns in pigs with fibroblast-laden bioinks. The approach improved wound healing, reduced scarring, and promoted angiogenesis while successfully demonstrating the complete intraoperative bioprinting workflow (Albouy et al., 2022).

#### 2.3.2 Handheld bioprinters

Handheld bioprinters represent a class of portable, user-friendly devices that operate *in situ* bioprinting environments through surgeon-operated manual printer control (Li et al., 2021).

A handheld skin printer was developed as a proof-of-concept approach capable of forming biomaterial and skin tissue sheets in murine and porcine excisional wound models. This approach demonstrated the ability to print to complex and different surfaces considering wound surfaces subject to different motions (Hakimi et al., 2018).

A more recent example of this technology is the BioPen, which allows for the direct writing of functional materials and is compatible with various bioinks for use in *in situ* bioprinting applications (Murphy and Atala, 2014) A similar approach was employed by using the SkinPen system to create gelation of bioink through direct application to skin surfaces which resulted in real-time bioink solidification (Zhou et al., 2023) A handheld bioprinting tool has also been developed for burn and skin wound healing that delivers living cells embedded within a biomaterial matrix which adapts to various wound shapes and surface angles up to 45° (Cheng et al., 2020).

In parallel, Yin and colleagues (Tianyuan et al., 2021) developed a bioprinter “gun,” a portable handheld device for *in situ* fabrication of composite wound dressings. This device integrates three material deposition techniques—spraying, extrusion, and electrospinning—within a single platform. This multifunctional material depositing enables the creation of personalized wound dressings that promote hemostasis, antibacterial protection, and tissue regeneration.

Another study by Ying et al. (2020) presented a low-cost, portable, open-source handheld bioprinter for *in situ* wound treatment. The battery-powered device features an ergonomic design and can directly print bioinks onto wounds to match their specific shapes. The researchers developed a new bioink which improves oxygen and liquid movement through tissue, thereby enhancing cell development and tissue regeneration. This method provides a successful personalized solution for fast wound dressing and healing.

The design of these portable tools requires strict adherence to ergonomic principles. Handheld devices often introduce challenges because attached cables can create obstacles which affect their balance and ability to move; therefore, designers create flexible attachments which help users maintain operational control. Effective ergonomic design creates products that match the natural hand shapes and motions of users. The most skilled fingers should operate precision controls while other fingers support stability needs and power requirements (Pazhouhnia et al., 2022) (Figure 2).

## 3 Integrating artificial intelligence with *in situ* 3D skin bioprinting

Artificial intelligence (AI) and machine learning (ML) can significantly enhance the performance and precision of bioprinting systems by ensuring consistency and reliability throughout the entire *in situ* 3D bioprinting workflow. This includes optimization of bioink formulation, automated image segmentation, control of the bioprinting process, and maintenance of printing quality. AI-based approaches also enable real-time detection of defects during and after printing, paving the way toward fully autonomous and intelligent bioprinting platforms. In the following section, some of these aspects are highlighted focusing on critical issues for future clinical translation (Ruberu et al., 2021; Sun et al., 2022).

### 3.1 Optimizing bioink formulation

*In situ* bioprinting requires bioinks that meet personalized needs while maintaining their physicochemical and biological properties. AI enables simultaneous optimization of bioink characteristics to improve structural stability and cell viability. These are some research works on bioink optimization.

Xu et al. (2025) developed an AI-guided framework that integrates Bayesian Optimization (Frazier, 2018) with Gaussian Process Regression (Deringer et al., 2021) to optimize bioink compositions for extrusion-based 3D bioprinting. The model predicted viscosity based on bioink composition, while Bayesian Optimization identified new formulations that maintained cell viability above 90% to achieve both printability and biological feasibility. Another group (Sarah et al., 2025) applied machine learning approaches to predict bioink viscosity through testing both material composition and shear forces. The study examined hybrid bioinks which contained different bioink components to determine their performance under various shear testing conditions.

A comprehensive investigation by Chen et al. (2023b) evaluated 210 bioink formulations using direct ink writing. They employed three machine learning (ML) algorithms to predict printability, all achieving over 80% accuracy. The study generated printability maps, or “printability windows,” to guide the development of new bioink formulations for biomedical 3D printing applications. In addition, Ruberu et al. (2021) developed an AI model to optimize bioink printability by measuring geometric accuracy between designed and printed constructs because bioink rheological properties determine printability (Gillispie et al., 2020) for GelMA and HAMA bioinks. The model used Bayesian optimization to discover optimal printing settings and bioink formulations which produced precise shapes with reduced testing requirements.

Furthermore, Oh et al. (2023) introduced a Rheology-Informed Hierarchical Machine Learning model which predicts bioink printing resolution based on various printing conditions and bioink compositions. The model demonstrated high accuracy for new, unseen bioinks. Lee et al. (2020) developed a machine learning–based approach to design naturally derived bioinks with high shape fidelity. Their analysis of mechanical properties showed that higher elastic modulus improves shape fidelity while extrusion stays possible until a specific yield stress limit. Zhang et al. (2024) predicted and optimized cell viability during extrusion-based 3D bioprinting by balancing print stability with protection against stress-induced cell damage. Their model predicted rheological parameters across different concentrations and temperatures, as well as cell survival under varying shear stress and exposure times.

To further advance bioink development, some models aim to generate synthetic or augmented datasets to improve model accuracy without needing extensive time-consuming experiments that incur high costs. One approach to generating such high-quality data was demonstrated by Schmieg et al. (2022) who enhanced quality control through MRI-based, non-destructive evaluation of hydrogel constructs. The researchers conducted a systematic evaluation of three different hydrogels and one reference material to assess their reproducibility by testing essential parameters which included porosity and void size and structural deviations throughout the scaffold height. The proposed methodology combines MRI-based structural assessment with comprehensive printing monitoring to establish a solid framework that enables standardized production processes.

### 3.2 Real-time monitoring and feedback loop

The quality of the bioprinting process is critical to ensure that the resulting printed tissues or organs possess appropriate structural and functional characteristics. Traditionally, the quality of the produced tissue has been evaluated through destructive tests such as mechanical testing, biological testing, and physical and chemical testing. However, to overcome the limitations of these post-production methods, scientists currently work on developing non-invasive methods for monitoring material quality and material behavior within biological systems (Kathirvel et al., 2024; Advincula et al., 2021).

The *in situ* bioprinting process is highly sophisticated, and all decisions should be carefully considered during it. To avoid excessively long operation times, the implementation of artificial intelligence (AI) can greatly enhance the feedback process. This involves image acquisition during the operation and analyzing it to support decision-making, assist physicians, and suggest appropriate printing parameters. Therefore,several approaches can be implemented in *in situ* bioprinting to enable easier and faster operations.

The first step is image acquisition. AI can facilitate real-time imaging and accuracy detection to monitor and evaluate the printing outcome. Yang et al. (2021) introduced the 3D Extrusion-Based Bioprinter-Associated Optical Coherence Tomography (3D P-OCT). This integration enables printers to optimize critical parameters during printing, such as filament size, layer thickness, and overall layer fidelity, ensuring high printing quality and providing physicians with valuable insights into the printed tissue.

As mentioned, the extended printing process duration results in bioink material changes because operational temperature and shear stress conditions shift which leads to changes in printing settings. Chen et al. (2022) developed the Artificial Intelligence–Assisted High-Throughput Printing-Condition-Screening System (AI-HTPCSS) to rapidly identify optimal bioprinting parameters. This system integrates a programmable pneumatic bioprinter capable of automatically adjusting parameters such as pressure, nozzle speed, and print distance, along with an AI-based image analysis algorithm that evaluates print fidelity and dimensional accuracy. The system generates a visual phase diagram which displays how different parameters impact print quality to provide a straightforward system for improving complex bioprinting procedures. A complementary approach was proposed by Webb and Doyle (2017) introduced the Printability Optimization Index as a standardized measurement system which evaluates print quality through both geometric precision and bioink shear stress testing. The researchers systematically varied nozzle size, pressure, and print speed to identify optimal, cell-friendly printing conditions.

To avoid unwanted issues during operation, AI can help detect hidden errors. The entire printed structure becomes compromised when additional droplets or unexpected bioink deposition occurs. To address this, Shi et al. (2019) employed machine learning to prevent satellite droplet formation (Shi et al., 2018). The model was trained to predict a droplet deformation parameter that indicates satellite formation using CFD data to optimize bioprinter voltage, bioink properties, and nozzle size to minimize satellite droplets while improving droplet speed and size.

Accurate control of bioink droplet volume is essential during droplet bioprinting for the precise deposition of biological materials and the replication of 3D microtissue environments. Shin et al. (2024), Shin et al. (2025) implemented machine learning (ML) and deep learning (DL) models to predict droplet volumes based on key bioprinting parameters, including bioink viscosity, nozzle size, printing time, printing pressure, and cell concentration.

Closed-loop control systems represent a new promising method that can enhance the ability to scale 3D bioprinting technology. Real-time monitoring in closed-loop 3D bioprinting is essential for precise print-head control and accurate bioink deposition (Zhu et al., 2021). In this respect, Islam et al. (2019) developed a lightweight, AI-driven cascaded convolutional neural network to segment robotic surgical instruments from high-resolution video with a prediction time of less than 6 min. Additionally, Haider et al. (2025) developed BioLite U-Net, which serves as a lightweight deep learning model that functions as a real-time monitoring tool within a closed-loop bioprinting system. It performs semantic segmentation on live images, identifying the nozzle, bioink, and background, which enables it to immediately detect and resolve printing errors. Post-operation, there is a need to evaluate the quality of cells inside the printed tissue to enhance and assess recovery (Margarita et al., 2025). Kim et al. (2021) evaluated a near-infrared (NIR) fluorescence imaging system with significant potential to non-invasively monitor transplanted cell-based tissue constructs in 3D bioprinting. They monitored cell movement within a 3D bioprinted tissue-engineered construct during live experiments. After implantation into a rat bone defect, the researchers used special cameras to capture NIR fluorescence from the implant which doctors used to trace living cells.

AI could soon enable non-invasive, motion-aware bioprinting by solving a key problem in minimally invasive surgery: organs constantly move and deform, making it extremely difficult to align preoperative scans with real-time laparoscopic video. Mountney and Yang (2010) addressed this issue by introducing an AI-driven computer-vision framework that simultaneously estimates camera motion and soft-tissue deformation directly from laparoscopic images. The system tracks organ motion with precision, which shows its usefulness for both autonomous surgical procedures and image-based surgical methods and bioprinting processes (Figure 3).

**FIGURE 3:**
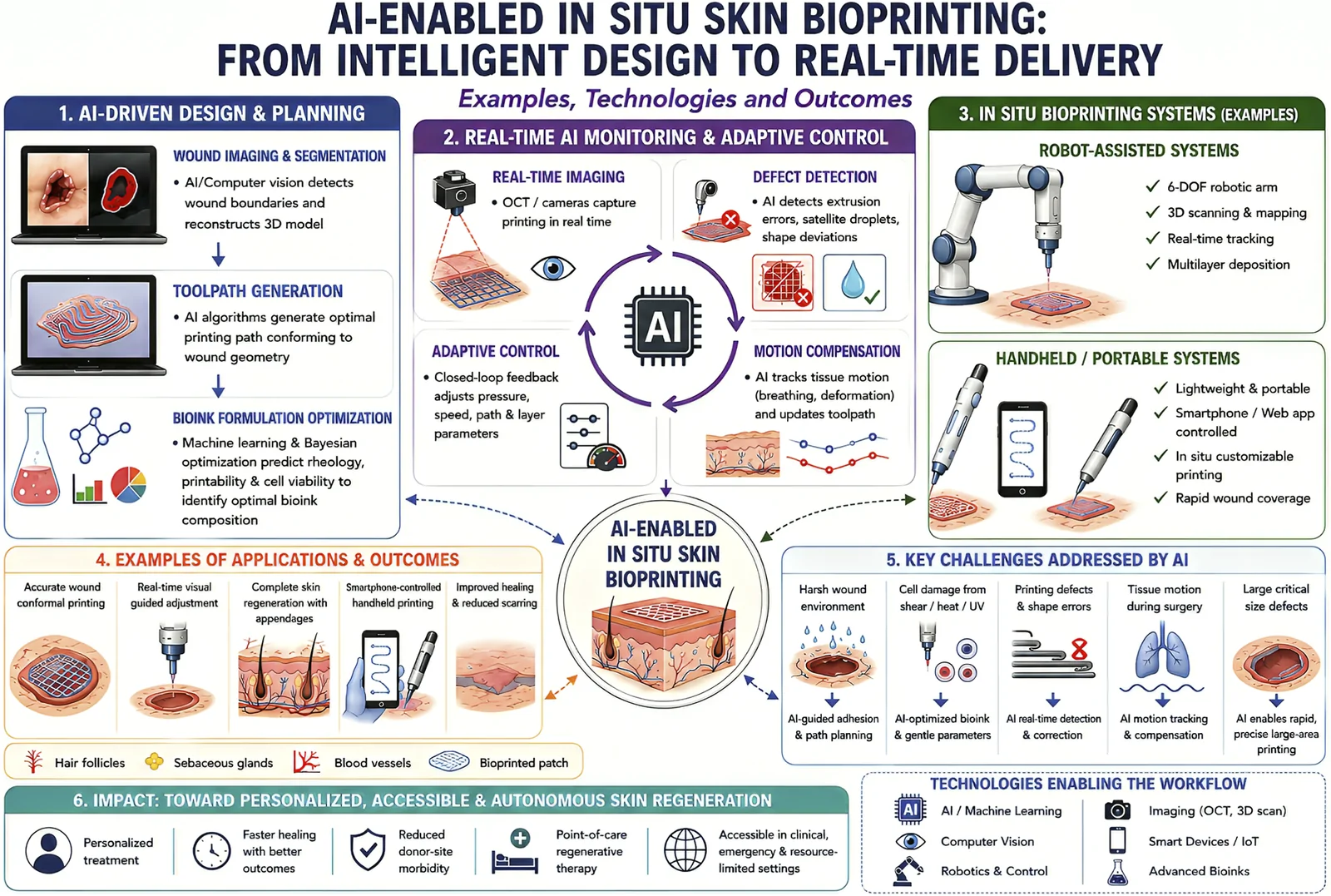
An illustrative summary diagram representing examples, technologies and potential outcomes of AI-enabled *in situ* skin bioprinting.

### 3.3 AI for *in situ* skin bioprinting

Some studies focus on AI usage during the *in situ* bioprinting process, and here we present some examples (Table 2).

**TABLE 2:** Skin *in situ* bioprinting studies that incorporated AI.

Author	System description	AI/Technology used	Bioink	Input data/Dataset size	Key finding/Result	Model validation	Clinical readiness (TRL)
Ding and Chang (2018)	• Image-guided platform combined surface imaging with automated toolpath generation to accurately match the wound shape for printing	• High-resolution surface imaging • Automated toolpath generation algorithm	gelatin (10% w/v) - alginate (1% w/v)	Surface topography scanned data	• Precise cell-laden construct printing within burn contours • maintain cell viability after 24 h	Wound-mimicking phantom (*Ex vivo*)	TRL 2 (*In vitro*/*ex vivo* proof-of-concept)
Gholami et al. (2018)	• Image segmentation-based system that detects chronic wound boundaries to guide bioprinting robot	• Image-based wound segmentation • G-code generation from wound contours	Alginate–gelatin hydrogel	26 wound images from 15 human subjects	• 95.56% correspondence between printed patch and intended wound geometry	26 wound images from 15 human subjects (proof-of-concept)	TRL 3 (Analytical and proof-of-concept validation using clinical data)
Jeong et al. (2024)	• INSIGHT system that uses image recognition to adjust the printing in real time • Multi-bioink printhead	• Computer vision that tracks wound boundaries • Real-time color and contour recognition	Multiple bioinks	Dynamic depth camera imaging and real-time visual streams	• Treated diabetic and trauma wounds • Dual print modes reduce printing time for large wounds	• *Ex vivo* porcine wound • *In vivo* diabetic/trauma models	TRL 4 (*In vivo* small/large animal validation)
Zhao et al. (2022b)	• Robot-assisted system with real-time tracking to enable printing operation	• Computer vision for wound detection • Real-time visual tracking • Automated path planning	Matrigel, Epidermal stem cells, Skin-derived precursor cells	Structured-light 3D surface point clouds	• Complete epidermis and dermis restoration • New hair follicles, sebaceous glands and blood vessels • Stable outcomes for 10 months	Full-thickness murine wounds (*in vivo*)	TRL 4 (*In vivo* preclinical animal validation)
Wang et al. (2024b)	• Smartphone-controlled programmable handheld bioprinter with Wi-Fi connectivity	• Smartphone-programmed path planning • Automated printing through instructions via Web App	Laponite XLG and HA/PEGDA granular hydrogel	Smartphone-captured wound images and programmed strip dimension inputs	• Precise strip dimensions and path programmed via smartphone • Uniform wound coverage with faster healing	• *Ex vivo* porcine skin • *In vivo* rat full-thickness skin wounds	TRL 4 (*In vivo* preclinical animal validation)

Ding and Chang (2018) developed a path planning model to advance *in situ* bioprinting for wound repair. The authors integrated high-resolution surface imaging with automated toolpath generation to produce deposition trajectories precisely matched to the wound’s geometry. The scanned surface data was processed to generate a customized print path, enabling direct bioprinting of a hydrogel construct that conformed accurately to the defect. Their results demonstrate that this imaging-guided workflow can reliably fabricate a graft replicating the topography of a wound-mimicking phantom.

Beyond this, Gholami et al. (2018) demonstrated image-guided wound-developing bioprinting with a proof-of-concept system for chronic wound treatment that integrates image-based wound segmentation with robotic bioprinting. The resulting wound contours were converted into G-code instructions for a bioprinter, which employed an alginate–gelatin hydrogel. The bioprinter successfully applied cell-laden hydrogel onto the wound surface, with a 95.56% correspondence between the printed patch and the intended wound geometry.

In a more innovative approach, Jeong et al. (2024) developed Intelligent *in situ* printing Guided by Eye-in-Hand robot Technology (INSIGHT), a cutting-edge computer vision-enabled system that integrates a depth camera with a 6-degree-of-freedom robotic arm capable of printing multiple bioinks simultaneously. This system empowers real-time adjustments and printing based on dynamic image recognition that distinguishes color and contour differences. The authors demonstrated its capabilities in *ex vivo* experiments on wounds in porcine models.

Zhao W. et al. (2022) established a robot-assisted *in situ* bioprinting system which they tested to create functional skin regeneration solution. The system combines advanced 3D scanning technology with real-time visual tracking capabilities and 6-DoF robotic movement control to enable its operation. Wounds on mice were precisely mapped using a structured-light 3D scanner, with conformal toolpaths generated for bioink deposition. The binocular eye-in-hand camera system tracked wounds with high precision (<0.8 mm error) to compensate for motion during printing. The printed constructs used to treat full-thickness murine wounds showed strong skin regeneration results which included complete epidermis and dermis restoration together with the development of new hair follicles and sebaceous glands and blood vessel formation, which remained stable for 10 months.

A new technology combines artificial intelligence with *in situ* skin bioprinting by creating a handheld bioprinter that can be programmed and controlled by a smartphone for use in dressing wounds. In this system, the strip dimensions and printing path are both precisely programmed and controlled through a smartphone interface, which guarantees accurate deposition and consistent print quality during *in situ* skin wound treatment (Wang C. et al., 2024) (Figure 3).

### 3.4 Next-generation AI paradigms in autonomous bioprinting

#### 3.4.1 Generalization challenges across the bedside bioprinting workflow

Despite recent progress, several challenges continue to limit the clinical translation of AI-assisted bioprinting. Predictive models remain constrained by limited and heterogeneous datasets, variability in bioinks and printing conditions, insufficient uncertainty quantification, and limited model interpretability, all of which reduce reliability and generalizability (Zhan et al., 2025; Bilbrey et al., 2025; Sui et al., 2026). Emerging AI technologies offer promising strategies to overcome these limitations and improve the robustness and clinical applicability of bioprinting systems.

#### 3.4.2 Digital twins for addressing data scarcity

Diffusion, crosslinking, and maturation processes can be predictively simulated using digital twin frameworks in bioprinting, allowing for parameter modifications prior to deviations. These systems offer a virtual testing field for new biomaterials or patient-specific grafts when combined with multi-omics and clinical datasets, significantly speeding up design-build-test cycles (Mueller et al., 2026). Large datasets, either from prior prints or from simulations (digital twins) of tissue growth and cell behavior, are usually used to train AI models that use machine learning. These models are then assessed on their capacity to generalize to new situations (Robazzi et al., 2026). The model’s predictions will be erroneous and produce less-than-ideal outcomes if the data is biased or incomplete. Therefore, the successful application of ML in bioprinting depends on the calibration and precision of data-collecting instruments, such as sensors or other hardware (Robazzi et al., 2026).

#### 3.4.3 Physics-guided priors and uncertainty quantification for materials and printing parameters

Model ensembling is a prominent method for quantifying uncertainty in order to find poorly learned or out-of-domain structures for active learning (Bilbrey et al., 2025). Although recent studies in materials modeling suggested calibrated prediction intervals, ensemble strategies, and physics-guided priors to flag out of distribution conditions during optimization and control, community analyses identified data sparsity, heterogeneous measurement protocols, and representation ambiguity as fundamental barriers to reliable generalization in polymers and gels, and uncertainty quantification remained underutilized in hydrogel applications (Zhan et al., 2025).

#### 3.4.4 Explainable AI (XAI) frameworks for clinical translation and decision transparency

To address the limited interpretability of deep learning models, it is essential to understand the underlying mechanisms driving model predictions rather than treating them as “black boxes.” Explainable AI (XAI) provides this capability by linking AI-identified patterns to biologically relevant features, thereby improving model transparency and supporting scientific validation (Sui et al., 2026). Two common methods are used to give input-output process relationships contextual logic, which are LIME (Local Interpretable Model-agnostic Explanations) and SHAP (SHapley Additive exPlanations) (Mueller et al., 2026). When applied to bioprinted constructions, for instance, an explainable AI framework might show that pore size distribution is one of the most important indicators of cell viability and differentiation outcomes. Smaller pore diameters are consistently linked to lower viability in particular areas of the construct, which could be highlighted by feature attribution techniques like SHAP. For example, the model can quantitatively map locations with essential pore sizes below oxygen diffusion thresholds to projected hypoxic zones, allowing for a direct comparison with oxygen gradients obtained experimentally. This realization can then be interpreted in the context of established biological mechanisms, such as the stabilization of HIF-1α in confined microenvironments due to hypoxia, where restricted oxygen transport results in altered metabolic states and compromised stem cell activity. In this sense, XAI offers mechanistic hints that link structural design factors to functional biological responses rather than just finding correlations. In the end, these interpretable correlations can guide logical scaffold design, allowing engineers to modify pore shape or vascularization techniques to preserve functioning stem cell niches and maintain oxygen homeostasis (Sui et al., 2026).

Ultimately, AI has the potential to support all stages of the *in situ* bioprinting workflow, from patient assessment and treatment planning to bioink selection, robotic bioprinting, and postoperative monitoring. By enabling continuous data analysis and closed-loop feedback, AI can enhance precision, personalization, and clinical decision-making. The complete AI-powered operating room workflow is illustrated in Figure 4.

**FIGURE 4:**
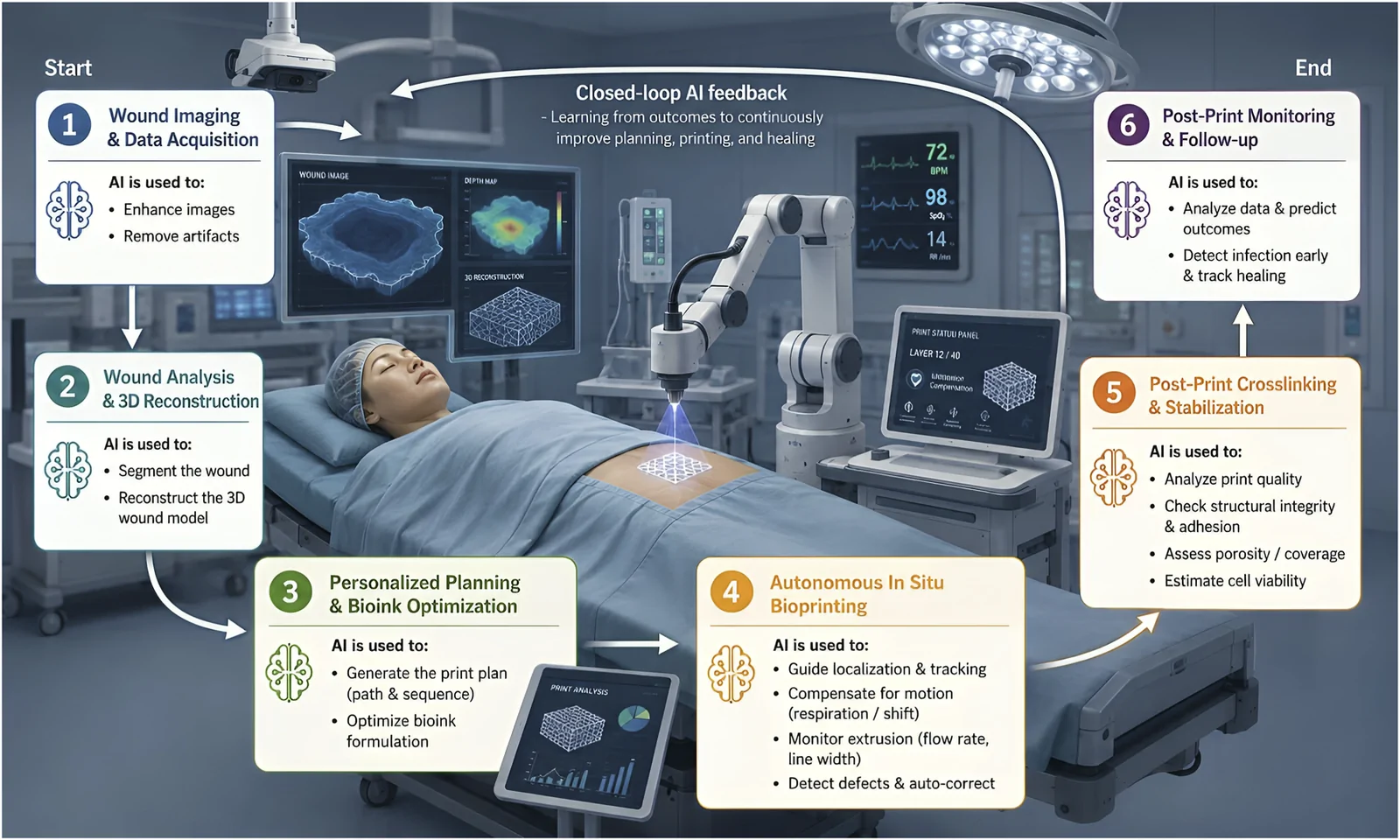
Illustrates an end-to-end, “bedside to bedside” clinical workflow for treating complex wounds using autonomous, *in situ* (on-patient) bioprinting, augmented by a suite of artificial intelligence (AI) technologies. The process is organized into six primary stages, interconnected by a closed-loop data feedback system that informs and optimizes subsequent treatments.

## 4 Translational, regulatory, and ethical governance for clinical translation

Translating 3D and AI-driven bioprinting from bench-scale research to routine clinical applications requires establishing robust governance, standardized data architectures, and harmonized regulatory frameworks (Mueller et al., 2026; Kantaros et al., 2025). Moving beyond early, fluid-phase innovations (technology readiness level-TRL 1–3) demands structured, evidence-based frameworks to systematically evaluate product safety, validate “*in vivo*” behavior, and manage the “effectiveness–economy” dilemma inherent in personalized design (O’Connell et al., 2024; Zhang et al., 2025). Navigating this transition relies on upstream engagement among scientists, ethicists, regulatory bodies, and clinicians to establish rigorous quality controls, safeguard intellectual property (IP), and ensure equitable global access to advanced regenerative therapies (Robazzi et al., 2026; Kantaros et al., 2025; Datta et al., 2023).

### 4.1 Regulatory approval pathways and translational hurdles

The regulatory oversight of 3D bioprinted products (BPPs) is fundamentally dictated by their structural and biological composition (Mueller et al., 2026; Zhang et al., 2025). In the United States, acellular constructs are categorized as medical devices under the food and drug administration; Center for Devices and Radiological Health (FDA CDRH) (e.g., 510(k) or PMA pathways), whereas cell-laden or growth factor-infused constructs fall under Center for Biologics Evaluation and Research (CBER) as human cells, tissues, or cellular/tissue-based products (HCT/Ps) requiring Biologics License Applications (BLAs) or Investigational New Drug (IND) submissions (Mueller et al., 2026). Similarly, in Europe, regulation bifurcates between the Medical Device Regulation (MDR) and the European Medicines Agency (EMA) Advanced Therapy Medicinal Products (ATMP) frameworks, alongside frameworks like the US 21st Century Cures Act (Mueller et al., 2026; Kantaros et al., 2025; Zhang et al., 2025). When artificial intelligence (AI) and machine learning (ML) are embedded into the bioprinting workflow, software functions are regulated under Software as a Medical Device (SaMD/SiMD) guidelines, guided by risk-based premarket reviews, the FDA’s AI/ML Action Plan, Good Machine Learning Practice (GMLP), and Predetermined Change Control Plans (PCCPs) to manage continuously learning or adaptive algorithms (Mueller et al., 2026; Robazzi et al., 2026).

Key translational hurdles stem from the lack of clear-cut regulatory predicates, unaligned global standards, and ambiguous guidelines for point-of-care or intraoperative “*in situ*” bioprinting (Mueller et al., 2026; O’Connell et al., 2024; Zhang et al., 2025). From an intellectual property (IP) and legal standpoint, proving the novelty and non-obviousness of bioprinted tissues without conflicting with the legal exclusion of “products of nature” remains challenging (Kantaros et al., 2025). Furthermore, cross-border clinical deployment is severely complicated by regional disparities in IP enforcement—ranging from established systems in the US, EU, Japan, and South Korea to fragmented infrastructures in developing nations (Kantaros et al., 2025). Regulatory compliance is further strained by the poor training reproducibility of AI models, lack of standardized evaluation metrics, and scenario-specific ML models (Sui et al., 2026; Zhang et al., 2025). Addressing these challenges requires defining a comprehensive regulatory strategy early (suggested at TRL 4)—encompassing classification, preclinical/clinical development, documentation, and post-market surveillance—while establishing universal ML models, reducing multi-source variability, and encouraging active researcher participation in global policy formulation (O’Connell et al., 2024; Zhang et al., 2025).

### 4.2 GMP manufacturing and technology readiness

Transitioning bioprinted constructs to clinical readiness requires establishing AI-assisted Good Manufacturing Practice (GMP/cGMP) facilities and standardized Quality Management Systems (QMS), such as ISO 13485 or FDA Quality System Regulations (Mueller et al., 2026; O’Connell et al., 2024). Industrial-scale scaling mandates moving away from research-grade components to medical-grade, cGMP-certified bioinks and raw materials complying with ISO 10993 biocompatibility standards (Mueller et al., 2026; O’Connell et al., 2024). To overcome the scalability limitations and high costs of manual laboratory operations, biomanufacturing platforms must adopt automated, “in-line” process control anchored in Quality by Design (QbD) principles, which systematically link Critical Process Parameters (CPPs) and Critical Material Attributes (CMAs) to Critical Quality Attributes (CQAs) (Zhang et al., 2025).

Achieving high Technology Readiness Levels (TRLs) depends on deploying multi-scale and multi-modal sensing arrays (e.g., CT, MRI, optical coherence tomography [OCT], infrared, and acoustic sensors) for continuous quality assurance (Zhang et al., 2025). Integrating digital twin simulations, reinforcement learning-based closed-loop controls, and self-driving laboratories enables real-time error correction, automated defect detection, predictive maintenance, and optimized multi-axis toolpaths (Sui et al., 2026; Mueller et al., 2026; Zhang et al., 2025). These automated workflows ensure batch-to-batch reproducibility, aseptic processing, validated porosity, and mechanical stability while minimizing bioink and cell waste (Sui et al., 2026; Mueller et al., 2026; Zhang et al., 2025). Additionally, technology readiness demands defining clear patent scopes for hardware mechanisms, bioink formulations, and scaffold synthesis methods, alongside establishing joint ownership agreements for multidisciplinary teams and balancing open-source collaboration with proprietary component protection to maintain commercial viability (Kantaros et al., 2025).

### 4.3 Ethics, patient data privacy, and clinician acceptance

The clinical deployment of AI-driven bioprinting introduces complex bioethical, data security, and social governance imperatives (O’Connell et al., 2024; Datta et al., 2023). Utilizing patient-specific anatomical and genetic data to construct digital bio-CAD models mandates strict compliance with privacy regulations, such as Health Insurance Portability and Accountability Act (HIPAA) and General Data Protection Regulation (GDPR) (Mueller et al., 2026; Kantaros et al., 2025; Zhang et al., 2025). Safeguarding sensitive genomic and radiological datasets during storage and cloud-based toolpath monitoring requires advanced technological controls, including homomorphic encryption, role-based access, federated learning, digital rights management (DRM), watermarking, and decentralized blockchain tracking to prevent data breaches, unauthorized G-code manipulation, or IP infringement (Mueller et al., 2026; Kantaros et al., 2025; Zhang et al., 2025).

Ethically, bioprinting raises fundamental concerns regarding donor cell sourcing (autologous, allogeneic, or stem cells), bodily autonomy, biological ownership rights, and long-term biological risks such as cell-type toxicity, immunogenicity, or tumorigenicity (Kantaros et al., 2025; Datta et al., 2023). Obtaining valid informed consent requires transparent communication regarding cell/material origins and active mitigation of public and media hype that inflates expectations regarding technology maturity (O’Connell et al., 2024; Datta et al., 2023). Exaggerated clinical claims pose severe ethical risks by misinforming vulnerable patients and skewing decision-making in clinical trials (O’Connell et al., 2024; Datta et al., 2023).

Furthermore, ethical governance must address major justice considerations: the immense capital costs of specialized machinery, trained personnel, GMP infrastructure, and expensive xenogeneic-free growth media threaten to deepen healthcare inequities, creating a divide between wealthy populations and resource-limited regions (Sui et al., 2026; Kantaros et al., 2025; Datta et al., 2023). Broader societal concerns also encompass religious or cultural reservations regarding cell sources, patent infringement liability among designers and healthcare providers, and the ethical implications of fabricating non-therapeutic functional enhancements beyond natural human baselines (Kantaros et al., 2025; Datta et al., 2023). Ultimately, securing clinician acceptance depends on resolving “black box” AI opacity through Explainable AI (XAI) tools—such as SHAP and LIME—which demonstrate algorithmic reliability, foster professional trust, and preserve a strict human-in-the-loop paradigm where surgical teams retain ultimate override authority (Sui et al., 2026; Mueller et al., 2026) (Table 3).

**TABLE 3:** Strategic Pathways for Clinical Translation, Regulatory Compliance, and Governance in AI-Enabled Bioprinting. Summary of core technological, regulatory, and bioethical bottlenecks alongside proposed solutions and strategic translational pathways required for bedside deployment.

Strategic domain	Identified problem/Translational bottleneck	Proposed solution and strategic pathway
Regulatory Approval Pathways	Complex product categorization across jurisdictions (acellular devices vs. cell-laden HCT/Ps/ATMPs) and lack of predicates for intraoperative *in situ* printing	Implement early regulatory planning (at TRL 4) to define preclinical/clinical strategies, engage in international policy harmonization, and adopt standardized submission blueprints
AI Algorithmic Oversight	Unpredictability and regulatory uncertainty surrounding dynamic, continuously learning AI models updating intraoperatively	Establish Predetermined Change Control Plans (PCCPs) and adhere to Good Machine Learning Practice (GMLP) under Software as a Medical Device (SaMD) frameworks
GMP and Quality Assurance	High batch-to-batch variability, contamination risks, and reliance on academic/research-grade materials	Transition early to medical-grade, cGMP-certified, xenogeneic-free bioinks complying with ISO 10993 standards and establish formalized QMS (ISO 13485)
Manufacturing Scalability	Labor-intensive, manual operations leading to slow production, high costs, and poor repeatability	Implement Quality by Design (QbD) with multi-sensor closed-loop control, digital twins, and self-driving automated biomanufacturing platforms
Intellectual Property (IP)	Ambiguity regarding patent eligibility for “products of nature,” overlapping multi-stakeholder claims, and conflict with open-source models	Establish clear IP guidelines defining novelty for modified constructs, implement multi-party joint ownership agreements, and utilize hybrid/tiered licensing models
Data Privacy and Cybersecurity	Vulnerability of sensitive patient genetic data and 3D anatomical scans to cyber-threats during cloud toolpath streaming	Enforce HIPAA/GDPR compliance through end-to-end encryption, federated learning, digital rights management (DRM), watermarking, and blockchain tracking
Bioethics and Informed Consent	Unrealistic patient expectations and skewed informed consent caused by exaggerated public/media hype surrounding technology maturity	Maintain transparent public communication regarding cell/material origins, active hype mitigation by researchers, and dynamic consent models
Healthcare Equity and Justice	High financial barriers (equipment, cGMP infrastructure, media) threatening to exacerbate disparities between wealthy and resource-limited regions	Develop cost-effective portable/handheld systems, establish tiered pricing structures, and adopt non-commercial licensing options for underserved global settings
Clinician Acceptance	Hesitancy among medical professionals to trust “black-box” AI decision-making during intraoperative toolpath generation	Integrate Explainable AI (XAI) feature-attribution tools (e.g., SHAP, LIME) and enforce a strict human-in-the-loop framework with surgical override authority

## 5 Conclusion

Directly bioprinting skin substitutes onto a patient’s wound bed offers a transformative paradigm for managing severe, extensive cutaneous defects, yet clinical translation is heavily constrained by real-world logistical and physiological hurdles. Moving out of controlled benchtop environments requires overcoming hostile, wet, and irregular wound topographies, dynamic tissue shifts caused by respiration or muscle motion, and critical cell viability loss during extended operational windows. Integrating data-driven automation addresses these bottlenecks directly by replacing manual trial-and-error with computational precision—leveraging machine learning for pre-operative bioink formulation optimization and computer vision for real-time motion tracking and print-head alignment during deposition.

To bridge the gap between benchtop concept and autonomous bedside delivery, our proposed clinical blueprint outlines four foundational strategic priorities:

1. Bioink Standardization: Transitioning to certified, clinical-grade (GMP) biomaterials validated under rigorous ASTM/ISO benchmarks.
2. Adaptive Closed-Loop Control: Deploying explainable AI architectures—integrating patient-specific Digital Twins and real-time optical coherence tomography (OCT) monitoring—to maintain dynamic error correction under direct surgical oversight.
3. Regulatory Governance: Establishing dedicated Software as a Medical Device (SaMD) pathways tailored to adaptive intraoperative software.
4. Hardware Democratization: Scaling flexible hardware onto ergonomic, portable handheld devices and multi-axis robotic arms suitable for both specialized operating suites and point-of-care emergency settings.

By systematically coupling adaptive AI software controls with standardized manufacturing and regulatory governance, *in situ* skin bioprinting can transcend historical donor graft constraints to deliver automated, patient-specific wound care globally.

## Glossary

AI: Artificial Intelligence
AI-HTPCSS: Artificial Intelligence–Assisted High-Throughput Printing-Condition-Screening System
Alg-Gel: Alginate–Gelatin
ATMP: Advanced Therapy Medicinal Product
cGMP: Current Good Manufacturing Practice
CFD: Computational Fluid Dynamics
CT: Computed Tomography
DBB: Droplet-Based Bioprinting
DL: Deep Learning
DRM: Digital Rights Management
EBB: Extrusion-Based Bioprinting
Epi-SCs: Epidermal Stem Cells
GelMA: Gelatin Methacrylate
GMLP: Good Machine Learning Practice
GMP: Good Manufacturing Practice
GDPR: General Data Protection Regulation
HAMA: Hyaluronic Acid Methacrylate
HCT/Ps: Human Cells, Tissues, and Cellular and Tissue-Based Products
HIPAA: Health Insurance Portability and Accountability Act
INSIGHT: Intelligent *in situ* Printing Guided by Eye-in-Hand Robot Technology
IP: Intellectual Property
ISO: International Organization for Standardization
LAP: Lithium Phenyl-2,4,6-Trimethylbenzoylphosphinate
LBB: Laser-Based Bioprinting
LIME: Local Interpretable Model-agnostic Explanations
ML: Machine Learning
MRI: Magnetic Resonance Imaging
NIR: Near-Infrared
OCT: Optical Coherence Tomography
PCCP: Predetermined Change Control Plan
PID: Proportional–Integral–Derivative
QbD: Quality by Design
QMS: Quality Management System
SaMD: Software as a Medical Device
SCARA: Selective Compliance Articulated Robot Arm
SHAP: SHapley Additive exPlanations
SKPs: Skin-Derived Precursors
TRL: Technology Readiness Level
UV: Ultraviolet
XAI: Explainable Artificial Intelligence
